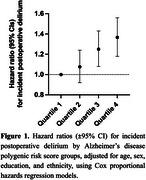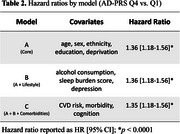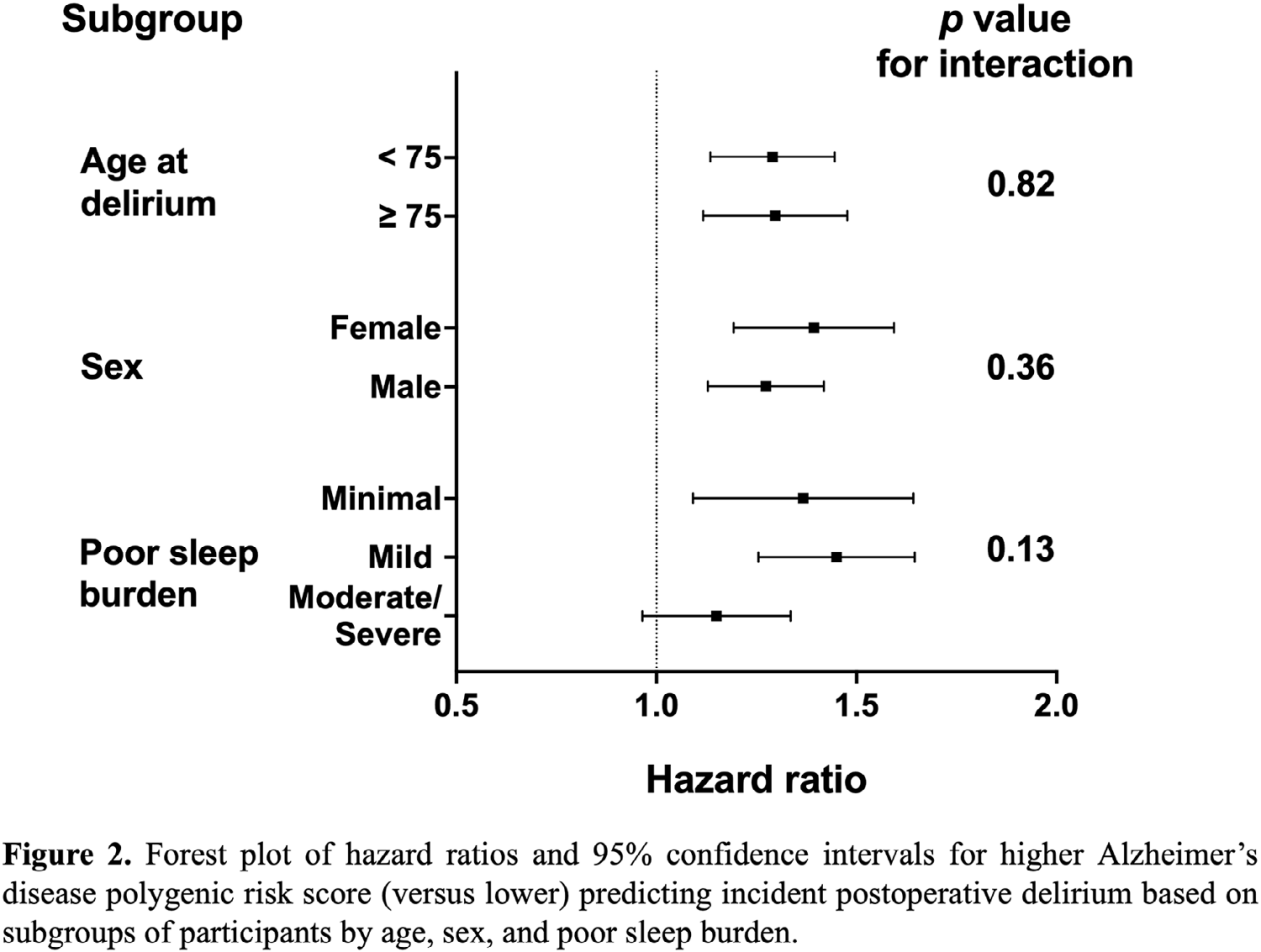# Associations between Alzheimer's disease polygenic risk and postoperative delirium: a prospective cohort study

**DOI:** 10.1002/alz70860_101679

**Published:** 2025-12-23

**Authors:** Yun Jin Chen, Arlen Gaba, Peng Li, Kun Hu, Hui‐Wen Yang, Lei Gao

**Affiliations:** ^1^ Queen's University School of Medicine, Kingston, ON, Canada; ^2^ Perioperative Sleep and Brain Health Lab, Massachusetts General Hospital, Boston, MA, USA; ^3^ Tufts Medical Center, Boston, MA, USA; ^4^ Massachusetts General Hospital, Boston, MA, USA; ^5^ Harvard Medical School, Boston, MA, USA; ^6^ Tzu Chi University, Hualien City, Hualien County, Taiwan

## Abstract

**Background:**

Postoperative delirium (POD), marked by acute mental status changes and impaired attention, affects up to one‐third of surgical patients, leading to significant morbidity, mortality, cognitive decline, and potential progression to Alzheimer's disease (AD). While *APOE‐ε4* is a recognized genetic risk factor for AD, its association with POD remains inconsistent. This study investigates whether a higher AD Polygenic Risk Score (AD‐PRS) predicts increased POD risk in patients without known dementia. Given the link between sleep disturbances, AD, and POD, we also examine how sleep modifies this relationship.

**Method:**

This prospective cohort study used data from 500,000 UK Biobank participants (mean age 57±8 years) to identify new‐onset delirium over a 12‐year median follow‐up. POD cases were identified using the International Classification of Disease‐10 coding, occurring within three days of surgery. Participants with pre‐existing dementia or dementia diagnosed within one year of POD were excluded. AD‐PRS was calculated as a weighted sum of genetic variants, with scores divided into quartiles due to the absence of standardized thresholds. Covariates included demographics, comorbidities, and lifestyle factors, with sleep burden categorized as minimal, mild, or moderate/severe. Cox proportional hazard models were used to evaluate the relationship between AD‐PRS and POD risk.

**Result:**

AD‐PRS was available for 487,269 participants, with 1610 cases of POD identified after excluding those with known dementia. A stepwise increase in POD risk was observed with higher AD‐PRS quartiles (Q). Compared to Q1, individuals in Q3 (Hazard Ratio = 1.23, 95% Confidence Interval [1.07–1.42], *p* < .01) and Q4 (1.35, [1.18–1.56], *p* < .001) had progressively higher POD risk (Figure 1). This association remained consistent in the fully adjusted model (Table 2). AD‐PRS was equally predictive for POD risk across the three poor sleep burden groups, sexes, and age groups (Figure 2).

**Conclusion:**

Higher AD‐PRS is independently associated with an increased risk of POD in adults without dementia. The predictive value of AD‐PRS remained consistent across sleep burden levels, sexes, and age groups. These findings highlight the role of polygenic Alzheimer's risk, beyond *APOE‐ε4*, in delirium susceptibility after surgery, but prospective surgical cohorts are needed to confirm these biobank findings.